# Serum and Urinary NGAL in Septic Newborns

**DOI:** 10.1155/2014/717318

**Published:** 2014-01-21

**Authors:** Mike Smertka, Jolanta Wroblewska, Anna Suchojad, Malgorzata Majcherczyk, Danuta Jadamus-Niebroj, Teresa Owsianka-Podlesny, Aniceta Brzozowska, Iwona Maruniak-Chudek

**Affiliations:** ^1^Department of Neonatal Intensive Care, Upper Silesian Centre of Child's Health, Medical University of Silesia, Ul. Medykow 16, 40-752 Katowice, Poland; ^2^Department of Pathophysiology, Medical University of Silesia, Ul. Medykow 18, 40-752 Katowice, Poland

## Abstract

Neutrophil gelatinase-associated lipocalin (NGAL) is postulated to be a potentially new and highly specific/sensitive marker of acute kidney injury (AKI). The aim of this study was to assess the impact of inflammation on serum and urine NGAL in newborns that were treated due to infection. We determined serum and urine NGAL concentrations in 73 infants (51 with sepsis; 22 with severe sepsis) admitted to the Intensive Care Unit in the first month of life, for three consecutive days during the course of treatment for infection. 29 neonates without infection served as the control group. Septic patients, in particular, severe sepsis patients, had increased serum and urinary NGAL levels in the three subsequent days of observation. Five septic patients who developed AKI had elevated serum and urinary NGAL values to a similar extent as septic neonates without AKI. A strong correlation was found between the concentration of serum and urinary NGAL and inflammatory markers, such as CRP and procalcitonin. Serum and urinary NGAL levels were also significantly associated with NTISS (neonatal therapeutic intervention scoring system) values. We conclude that increased serum and urinary NGAL values are not solely a marker of AKI, and more accurately reflect the severity of inflammatory status.

## 1. Introduction

Sepsis is recognized as one of the most severe pathologies in newborns and young infants, responsible for almost one and a half million deaths each year, worldwide. Severe sepsis and septic shock may lead to (multi)organ dysfunction syndrome, including acute kidney injury. AKI is regarded as a highly negative prognostic marker. The epidemiological data in newborn patients is unknown, but clinical observation implies that it is a frequent occurrence.

Clinical symptoms suggesting acute kidney injury (AKI) occur late, but the pathology may quickly progress to acute renal failure. Currently the diagnosis of AKI is based on the increased levels of serum creatinine using serial measurements in addition to oliguria [1].

As neonates commonly develop nonoliguric AKI, Koralkar et al. proposed modification of the AKIN definition and does not include urine output as a diagnostic criterion in neonates [2]. The early diagnosis of AKI is crucial in the initiation of therapy; however, the assessment of decreased kidney function on the basis of serial creatinine measurements is delayed [3]. Serum creatinine as a functional kidney marker does not indicate kidney tissue injury; it only measures the accumulation of the endogenous marker as a consequence of decreased glomerular filtration rate (GFR). Furthermore, in newborns on their first days after delivery, serum creatinine reflects placental transfer of creatinine (maternal component). As such, an elevation of serum creatinine is commonly observed in the first week of life, and it usually declines over time.

New markers of AKI, for example, NGAL (neutrophil gelatinase-associated lipocalin), KIM-1 (kidney injury molecule 1), and IL-18 (interleukin 18), evaluated both in serum or urine, have an advantage over serum creatinine by directly reflecting injury to the kidney. NGAL, known as lipocalin 2, and initially described as an oncogene expressed by multiple human cells, including epithelial cells and neutrophils [4]. The major biological role of NGAL is its bacteriostatic feature related to iron-chelating properties [5]. NGAL, a 24-kDa protein, is rapidly eliminated from circulation by the kidney. The half-life of NGAL is estimated at 10–20 minutes [6]. It is postulated that increased serum and urinary NGAL are a sensitive marker of AKI, predominantly of damage to tubular cells, but do not reflect the decline of GFR.

The assessment of urinary NGAL in comparison to serum measurement seems to be more specific to kidney injury. It was reported in adults that lower respiratory tract infections are followed by the increase of serum NGAL, while urinary tract infections were the cause of high urinary NGAL [7, 8]. The data concerning AKI in neonates is sparse. Lavery et al. [9] demonstrated an inverse relation between body weight and urinary NGAL in preterm infants, while urinary NGAL was found to be elevated in children with congenital obstructive urinary tract anomalies [10].

The aim of this study was to assess the impact of inflammation on serum and urine NGAL in septic newborns.

## 2. Material and Methods

One hundred and two neonates in the first month of life were enrolled. They were all out-born and admitted to the hospital from home via the hospital emergency department or transferred to the unit from other hospitals by specialized neonatal ambulance. The reasons for the admission were as follows: suspicion of early or late infection or disturbances in neonatal period (mainly respiratory distress, hyperbilirubinemia, and failure to thrive). Those, who were diagnosed with sepsis or localized infection (pneumonia, urinary tract infection) of mild course, created the sepsis group (*N* = 51). Other infected newborns, presenting with severe sepsis and/or septic shock, formed the severe sepsis group (*N* = 22). Newborns, observed in the NICU and declared as noninfective cases, constitute the control group (*N* = 29). Clinical evaluation of the study group is presented in the Table 1.

Inclusion criteria consisted of gestational age equal to or higher than 34 wks and signed formal consent by parents or legal guardians. Patients with malformation of the urinary system and confirmed severe perinatal asphyxia were not included in the observation. In general, the patients were born in good condition.

The study protocol was approved by the Local Bioethics Committee (KNW/0022/KB1/120/11) and performed in a single third-level nursery (intensive care unit) of the University Children's Hospital. The protocol did not allow for any changes from the basic algorithmic diagnosis and treatment commonly accepted in the unit for the purpose of this study. All included patients were treated within the standards of care and the preservation of serum samples for further biochemical analysis was the only deviation.

All study participants were screened for microbiological colonization at admission and the basic septic screen was performed, which includes CBC (complete blood count) with blood smear, CRP (C-reactive protein), PCT (procalcitonin), glucose, electrolytes, and blood culture, as well as necessary biochemical evaluation including creatinine, total protein, serum albumin, and bilirubin. Urinalysis and urine cultures were also performed. Serum samples were collected, after the routine tests had been completed, and frozen to −70°C in polypropylene tubes.

The observational period started when the patient was admitted and underwent clinical evaluation (confirmation or exclusion of infection, investigation of the etiology of pathological symptoms) or when the patient during hospitalization presented clinically evident symptoms of infection and antibiotic therapy was introduced. The standard protocol included biochemical screening (glucose, creatinine, and aminotranspherases), CBC, and ABG (arterial blood gases) every day or more often, according to clinical requirements. During routine daily blood testing were performed, samples of serum and urine for NGAL evaluation were frozen; additionally, serum concentrations of cystatin C were evaluated and urine samples for albumin were saved.

### 2.1. Laboratory Measurements

Serum and urine total NGAL measurements were performed using commercially available ELISA kits (BioPorto Diagnostics Gentofte, Denmark) with intra- and interassay coefficients of variation of 3.6 and 7.9%, respectively (sensitivity below 0.01 ng/mL). Cystatin C measurements were performed by ELISA according to the manufacturer instruction (R&D, Minneapolis, MN, U.S.), with intra- and interassay coefficients of variation <5.9%. Albumin was evaluated by ELISA (Immundiagnostik AG, Bensheim, Germany), with intra- and interassay coefficients of variation of <5 and 8%, respectively.

### 2.2. Data Analysis

Sepsis was defined as SIRS and evidence of infection (positive microbiological culture, clinical symptoms). Severe sepsis was identified, when the course of sepsis was complicated by dysfunction of two or more organs or systems.

Estimated glomerular filtration rate (eGFR) was calculated according to the Schwartz formula. The estimates were not used for analysis of GFR changes as they were frequently calculated in unstable conditions on the first days of observation. Only serum creatinine values at discharge fulfilled steady-state conditions for GFR estimation.

AKI was defined as persistently increased serum creatinine (≥1.5 mg/dL) for at least 24 hours or rising values >0,3 mg/dL from the baseline [2].

### 2.3. Statistical Analysis

Analyses were performed using the STATISTICA 10.0 (StatSoft Polska, Kraków, Poland) software. Normality of distribution was tested with the Kolmogorov-Smirnov test. Presented data are expressed as median values with 1 and 3 quartiles or means with 95% confidence intervals. *U* Mann-Whitney test for comparison of independent variables and Friedman ANOVA for serial measurements were used. *χ* ^2^ test and *χ* ^2^ test with Yates's correction were used to compare distribution between groups. Correlation coefficients were calculated according to Spearman. *P* values < 0.05 were considered as statistically significant.

## 3. Results

At the time of enrolment, serum concentrations of creatinine and cystatin C were similar in neonates with sepsis, severe sepsis, and the control group (Table 1). Septic neonates were characterised by significantly increased levels of CRP and procalcitonin. In patients with severe sepsis platelet count was decreased. No difference in serum creatinine, cystatin C, and eGFR between study groups was observed. There was no decrease in urinary output in septic patients. Increased serum and urinary levels of NGAL were observed in septic patients, in particular with severe sepsis (Table 1).

On the basis of serum creatinine changes, the diagnosis of AKI was made only in 5 septic neonates. Only one AKI patient had low urinary output. There was no AKI patient that developed acute renal failure necessitating dialysis therapy.

During the 3-day observation period, serum creatinine declined in the control group and septic patients but not in the severe sepsis subgroup (Table 2). No changes in serum levels of cystatin C were found (Table 2). In the control group both serum and urinary NGAL levels were stable, while in septic and severe septic subgroups a decline in serum NGAL was observed with a 24-hour delay. The changes in urinary NGAL levels were not statistically significant.

**Figure 1:**
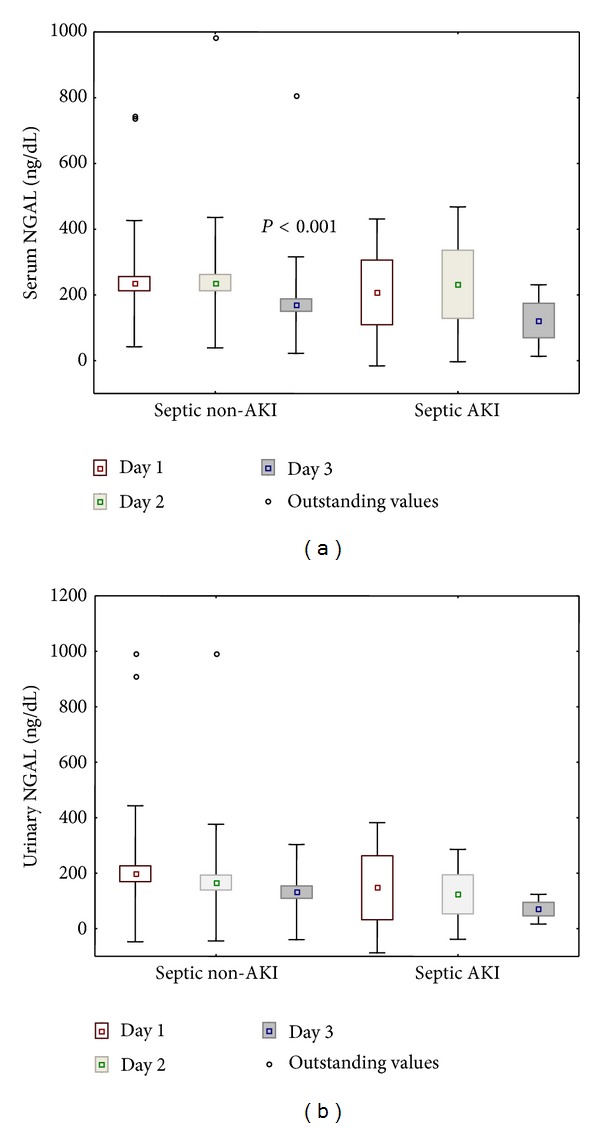
Serum and urinary NGAL concentrations in septic patients diagnosed with AKI (*N* = 5) and those who did not fulfill AKI definition (*N* = 68). Statistical significance versus day one.

Serum and urinary NGAL levels in septic patients who developed AKI were in a similar range to values observed in septic patients without AKI (Figure 1).

Septic patients with urinary tract infections were characterized by significantly increased urinary NGAL levels and markedly increased urinary/serum NGAL ratio (Table 3).

The duration of hospitalization was slightly, but not significantly, longer in both septic groups (Table 1). Only one patient died (with severe sepsis), and at discharge no difference in kidney function was observed between all the study groups eGFR; mL/min: 48,1 (41,0–55,2) in the control; 51,2; (47,8–54,7) in the sepsis group; and 47,9; (39,5–56,3) in the severe sepsis group.

### 3.1. Correlation Analyses

Analysis of demographic factors revealed the lack of correlation between serum and urinary NGAL, gestational age, birth weight, 5′ Apgar score, and gender. Both serum and urinary NGAL levels were correlated with NTISS values (Table 4).

There was a weak correlation between serum NGAL (significant at some points in time) and serum creatinine or eGFR, but not with serum cystatin C. No correlation between urinary output and both serum and urinary NGAL was found.

**Figure 2:**
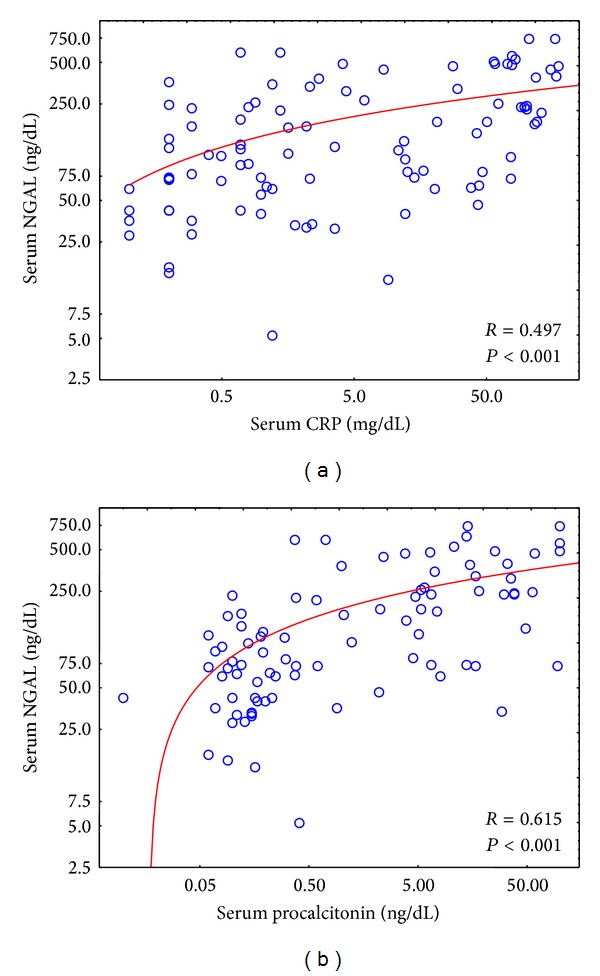
Correlations between serum CRP (a) and procalcitonin (b) and NGAL on the first day in the whole study group. All CRP, procalcitonin, and NGAL values were logarithmically transformed.

Significant correlations were noticed between the serum NGAL levels and CRP, and PCT, as well as WBC (white blood count), assessed during 3 subsequent days (Table 4, Figure 2). Similarly urinary NGAL levels correlated significantly with CRP and, PCT, and in some assessments, with WBC. Additionally, serum NGAL was inversely related to the platelet count (Table 4).

There was no correlation between serum and urinary NGAL values at admission and GFR at discharge. Serum NGAL levels at admission correlated with the duration of hospitalization (*R* = 0.222; *P* = 0.03). There was also a correlation between initial PCT values and the duration of hospitalization (*R* = 0.302; *P* = 0.006). Similar correlation with CRP values did not reach statistical significance (*R* = 0.177; *P* = 0.09).

## 4. Discussion 

The results of the study revealed that the measurement of NGAL cannot be solely evaluated as a marker of AKI in septic newborns. Equally elevated serum and urinary NGAL values were observed in septic newborns with and without AKI diagnosed on the basis of current criteria. Thus, the long-term search for the ideal marker of AKI has clearly not been completed yet. Moreover, our data suggest that measurement of NGAL, especially in serum, can be regarded as a marker of the severity of inflammatory status. This finding is corroborated by other studies showing NGAL elevation in pathologies thought to be of inflammatory origin or of a multifactorial origin where inflammation is implicated, such as in bronchopulmonary dysplasia [11] and autoimmune vasculitis [12].

The search for AKI markers is based on the poor prognosis of AKI in newborns, partially caused by the late diagnosis and therapeutic intervention. Diagnosis made only on the basis of serum creatinine level is burdened by error related to maternal serum creatinine (on the few first days of life) and functional immaturity of the tubules [13]. Urine output is not very useful, as nonoliguric AKI is claimed to often be in this group of patients [3]. Among all AKI cases, the injury caused by sepsis seems to be diagnosed particularly late, with minimal possibility for timely treatment.

Recent years have offered several studies evaluating various markers, potentially throwing light on the kidney condition in some clinical situations, including sepsis. Great expectation has been linked with cystatin C, and in adult medicine it was perceived at least by some investigators as alternative to creatinine marker. There are few authors reporting on cystatin C concentrations during the first days of life [14–18] and the claim is that cystatin C is an effective and earlier surrogate marker of decreased GFR than plasma creatinine in unselected ICU population. Yet some authors are not very enthusiastic about its ability for quick and precise recognition of impaired kidney function in sepsis [19]. Our observations are unfortunately very similar [20]. The current study provides the same results.

NGAL has been initially introduced in patients undergoing cardiosurgery. The start of injury and duration of the process was relatively easy to estimate, and that allows measuring the time between the incitement of injury and the rise in NGAL urine concentration [21]. Some promising results have been presented by Bennett et al. [22], who claimed that severity of kidney injury after cardiac surgery can be predicted by urinary NGAL analysis.

In our study, serum and urinary NGAL concentrations strongly correlate with inflammatory markers, for example, CRP and PCT. Inflammation is a recognized risk factor for AKI, but it does not reflect kidney injury, which was not frequently found in our study group. On the basis of the AKIN definition (serum creatinine equal to or above 1.5 mg/dL, or its increase for at least 0.3 mg/dL), AKI was diagnosed only in 5 patients. The described findings do not allow for the entire exclusion of sub-AKI in other patients, but they do not provide any additional arguments for the damage. Our results strongly pointed to NGAL as a marker of endothelial injury and neutrophil activation, rather than solely AKI, particularly in septic patients. Our results are in line with recently published papers [23–27] All of these authors recently described increases in serum NGAL in septic patients without correlation to AKI. In turn, each attempted to find a way to use other biomarkers in conjunction with both serum and urinary NGAL in order to accurately identify patients with both sepsis and AKI. These results add further credibility to the hypothesis that neither form of NGAL will be useful as a troponin-like cardiologic biomarker for AKI. As AKI is thought to be a multifactorial process, [28] it seems intuitive that adjunctive markers of detecting AKI in addition to NGAL will be population specific. (i.e., septic AKI, ischemic AKI related to oxygen delivery, contrast induced, other medical therapies, etc.) It also remains possible that, due to the variable and complex interactions causing AKI, efforts will be focused on identifying normal and abnormal physiologic parameters of these individual components, rather than the summation of their effects in order to detect and effect timely treatment. Such is evident in a similar manner to the limitations of current methods of AKI detection. Furthermore, it will need to be determined as to whether or not this increased testing is financially beneficial compared to more subjective methods currently employed or empirical treatment.

Obviously, we can see that the value of urine total NGAL measurements is evidently rising in newborns with urinary tract infection. However, using this parameter in sepsis is not demonstrating the evident answer. Unless definitely excluded, urinary tract infection has to always be suspected with elevated urine total NGAL values, making the estimation of AKI in systemic inflammatory response syndrome or infectious origin impossible. Our subgroup of patients with urinary tract infections was characterized as increased, compared to other septic patient levels, and a very high urinary/serum NGAL ratio. Using molecular methods for distinguishing the origin of NGAL (neutrophile versus tubular) might be helpful in solving this problem [29], but currently it would be difficult to be introduced into everyday clinical practice.

Perhaps serum NGAL should be evaluated as a marker of severity of systemic inflammatory response and its correlation with survival should be assessed. We have demonstrated that serum total NGAL is associated, though weakly (*R* = 0,222), with the length of hospitalization. However, recently, it was demonstrated that urinary NGAL is not a useful predictor of outcome in critically ill patients with risk of AKI [30]. In our study, both serum and urinary NGAL were not related to the renal outcome. There was no correlation between initial serum and urinary NGAL values and discharge. Knowing neutrophils are also the source of NGAL; it is quite possible that the inflammatory response, with increased release of neutrophils, will have the effect of higher serum total NGAL concentration. It would be interesting if urinary NGAL can be exclusively used for evaluation of the severity of urinary tract infection. Again however, in such cases, the systemic inflammatory reaction caused by sepsis must be excluded.

### 4.1. Limitation of the Study

The limitation of our study is the small number of AKI patients. As a consequence, the statistical power of this comparison between AKI and non-AKI patients is limited. It can be expected that, with larger AKI group, the difference in NGAL might possibly reach statistical significance. Therefore, we are unable to preclude any value of increased serum and urinary NGAL in AKI neonates. Additionally, an increase, especially in urinary NGAL levels, in septic patients may be indicative of inflammatory tubular injury without overt changes in GFR, the so-called subclinical kidney injury that is very difficult to detect and research. However, the increased value of this marker in septic patients without AKI decreases specificity and lowers its sensitivity in AKI detection. An additional limitation was the assessment of total NGAL using a commercially available kit. The assessment of monomeric NGAL secreted by injured kidney tubular cells and not by activated neutrophils could be more appropriate [31].

In conclusion, we suggest that increased serum and urinary NGAL values are not solely a marker of kidney injury in septic newborns and reflect the severity of inflammatory status.

## Figures and Tables

**Table 1:** Initial clinical characteristics of the study group (*N* = 102)—median values and 1–3 quartiles.

	Control group (*N* = 29)	Sepsis (*N* = 51)	Severe sepsis (*N* = 22)
Gender (m/f)	13/16	30/21	17/5*
Cesarean section (*N* (%))	11 (38%)	24 (47%)	10 (45%)
Gestational age (wks)	39 (37–39)	39 (38–40)	38 (36–40)
Births weight (g)	3150 (2850–3460)	3330 (2900–3570)	3110 (2610–3490)
Births length (cm)	53 (51–54)	54 (51–56)	52 (50–55)
Apgar 1 min (pts)	9 (9-10)	10 (7–10)	9 (7–10)
Apgar 5 min (pts)	10 (9-10)	10 (8–10)	9.5 (8–10)
Day of life (d)	6 (3–14)	7 (2–17)	9.5 (5–13)
Duration of hospitalization (d)	16 (12–21)	21 (17–25)	20 (17–23)
NTIS score (pts)	3 (2–5)	7 (4–9)^*∧*^	21 (12–29)^*∧*^
Leucocytes (10^9^/L)	11.2 (9.8–12.4)	15.0 (11.9–18.5)^*∧*^	13.5 (7.1–20.6)
Thrombocytes (10^6^/L)	267 (185–326)	266 (204–314)	165 (100–271)**
PCT (ng/mL)	0.13 (0.09–0.18)	1.82 (0.22–7.09)^*∧*^	10.60 (2.22–56.38)^*∧*^
CRP (mg/dL)	0.5 (0.2–1.0)	9.0 (1.1–42.0)^*∧*^	54.0 (36.0–100.5)^*∧*^
Creatinine (mg/dL)	0.79 (0.63–0.92)	0.76 (0.48–0.99)	0.85 (0.63–1.07)
eGFR (mL/min)	29.6 (27.8–37.9)	32.5 (24.8–51.1)	24.1 (21.1–44.1)
Cystatin C (mg/L)	1.72 (1.57–2.10)	1.54 (1.46–1.85)	1.65 (1.31–2.03)
Urinary output (mL/kg/h)	1.0 (0.8–1.3)	1.4 (1.2–1.6)*	1.4 (1.1–1.7)
AKI (*N* (%))	0	3 (5.9)	2 (9.1)
Urinary albuminin (mg/dL)	23.4 (15.4–47.7)	40.1 (29.2–56.8)	35.7 (26.1–48.8)
Serum NGAL (ng/mL)	69.7 (39.6–119.4)	153.5 (71.4–267.8)^*∧*^	228.6 (72.6–508.6)^*∧*^
Urinary NGAL (ng/mL)	20.6 (10.2–37.1)	59.0 (30.5–198.7)^*∧*^	131.2 (29.9–457.0)^*∧*^
Urinary/serum NGAL	0.30 (0.13–0.51)	0.37 (0.20–0.93)	0.50 (0.22–1.39)

Statistical significance versus control group. *P < 0.05; **P < 0.01; ^*∧*^ *P* < 0.001.

**Table 2:** The values of creatinine, cystatin C, and NGAL in three subgroups of newborns on the following time-points. Mean value, 95% CI, and statistical significance of differences between the subgroups A, B, and C (*U* Mann-Whitney test) are presented, together with intragroup changes (ANOVA test).

	Control group (*N* = 29) (A)	Sepsis (*N* = 51) (B)	Sever sepsis (*N* = 22) (C)	Statistical significance
Creatinine (mg/dL)				
Initial	0.81 (0.70–0.91)	0.76 (0.67–0.85)	0.86 (0.70–1.02)	NS
After 24 hours	0.74 (0.63–0.84)	0.74 (0.65–0.83)	0.87 (0.71–1.03)	NS
After 48 hours	0.67 (0.59–0.74)	0.63 (0.57–0.69)	0.89 (0.60–1.19)	NS
ANOVA	*P* < 0.001	*P* < 0.001	NS	

Cystatin C (mg/L)				
Initial	1.82 (1.68–1.96)	1.79 (1.57–2.01)	1.71 (1.40–2.01)	NS
After 24 hours	1.95 (1.64–2.26)	1.54 (1.42–1.67)	1.55 (1.22–1.89)	NS
After 48 hours	1.75 (1.59–1.90)	1.53 (1.42–1.64)	1.67 (1.32–2.02)	NS
ANOVA	NS	NS	NS	

sNGAL (ng/dL)				
Initial	109.9 (69.5–150.3)	203.8 (156.8–251.5)	296.5 (193.1–400.0)	C versus A *P* < 0.001 B versus A *P* < 0.001
After 24 hours	84.2 (53.8–114.6)	221.9 (160.2–283.6)	276.0 (178.2–373.8)	C versus A *P* < 0.001 B versus A *P* < 0.001
After 48 hours	83.8 (49.8–117.7)	151.8 (112.7–191.4)	202.5 (98.2–306.7)	C versus A *P* < 0.001 B versus A *P* < 0.05
ANOVA	NS	*P* < 0.001	*P* = 0.01	

uNGAL (ng/dL)				
Initial	40.4 (13.8–67.1)	157.5 (101.3–213.8)	286.6 (137.0–436.2)	C versus A *P* < 0.001 B versus A *P* < 0.001
After 24 hours	41.2 (5.8–76.5)	137.0 (85.0–189.0)	106.8 (57.3–156.3)	C versus A *P* < 0.001 B versus A *P* < 0.001
After 48 hours	46.4 (25.8–67.0)	106.8 (57.3–156.3)	177.4 (66.8–287.8)	C versus A *P* < 0.01 B versus A *P* < 0.05
ANOVA	NS	NS	NS	

**Table 3:** Serum and urinary NGAL levels in septic newborns with and without urinary tract infections on the following time points. Mean value, 95% CI, and statistical significance of differences between the subgroups (*U* Mann-Whitney test) are presented, together with intragroup changes (ANOVA test).

	Urinary tract infection (*N* = 17)	Septic patients without UTI (*N* = 54)	*P*
sNGAL (ng/dL)			
Initial	188.6 (102.1–275.0)	246.4 (192.0–300.7)	NS
After 24 hours	250.5 (108.8–392.3)	232.5 (178.9–286.1)	NS
After 48 hours	170.3 (76.8–263.8)	164.1 (119.7–208.6)	NS
ANOVA	NS	*P* < 0.001	

uNGAL (ng/dL)			
Initial	250.5 (108.8–392.3)	158.2 (91.0–225.4)	0.001
After 24 hours	275.7 (150.5–401.0)	126.7 (68.6–184.7)	0.003
After 48 hours	189.8 (69.5–310.2)	108.3 (58.5–158.2)	NS
ANOVA	0.07	NS	

uNGAL/sNGAL ratio			
Initial	4.35 (0.53–8.18)	0.59 (0.34–0.85)	<0.001
After 24 hours	2.58 (0.98–3.77)	0.52 (0.38–0.67)	<0.001
After 48 hours	1.07 (0.66–1.49)	0.66 (0.44–0.88)	0.04
ANOVA	NS	NS	

**Table 4:** Univariate correlations.

	NGAL					
	Day 1		Day 2		Day 3	
	Serum	Urinary	Serum	Urinary	Serum	Urinary
NTIS	*R* = 0.323	*R* = 0.241	*R* = 0.376	*R* = 0.292	*R* = 0.292	*R* = 0.228
	*P* = 0.001	*P* = 0.02	*P* < 0.001	*P* = 0.007	*P* = 0.008	*P* = 0.04
Creatinine	*R* = 0.278	NS	NS	NS	*R* = 0.317	*R* = 0.225
	*P* = 0.006				*P* = 0.004	*P* = 0.05
eGFR	*R* = −0.247	NS	NS	NS	*R* = −0.273	NS
	*P* = 0.02				*P* = 0.01	
Cystatin C	NS	NS	NS	NS	NS	NS
Urinary Output	NS	NS	NS	NS	NS	NS
WBC	*R* = 0.318	NS	*R* = 0.338	*R* = 0.250	*R* = 0.328	*R* = 0.275
	*P* = 0.001		*P* = 0.002	*P* = 0.03	*P* = 0.003	*P* = 0.01
PLT	*R* = −0.359	NS	*R* = −0.237	NS	*R* = −0.230	NS
	*P* < 0.001		*P* = 0.03		*P* = 0.04	
CRP	*R* = 0.497	*R* = 0.483	*R* = 0.703	*R* = 0.563	*R* = 0.649	*R* = 0.614
	*P* < 0.001	*P* < 0.001	*P* < 0.001	*P* < 0.001	*P* < 0.001	*P* < 0.001
PCT	*R* = 0.615	*R* = 0.343	*R* = 0.683	*R* = 0.489	*R* = 0.605	*R* = 0.462
	*P* < 0.001	*P* = 0.001	*P* < 0.001	*P* < 0.001	*P* < 0.001	*P* < 0.001
